# Accountable Government Spending: A Cross-National Analysis of Child Mortality in Developing Nations

**DOI:** 10.1177/0020731420960972

**Published:** 2020-10-05

**Authors:** Jamie M. Sommer

**Affiliations:** 1 Department of Sociology, University of South Florida, Tampa, Florida, USA

**Keywords:** development, child health, cross-national, governance, health

## Abstract

What can national governments do to improve their capacity for well-being? While increasing public medical care expenditures can facilitate increased well-being in developing nations, cross-national research often finds that public medical care expenditures have no effect on indicators of well-being, such as child mortality. This ineffective public spending could be due to a lack of governance; however, this relationship is understudied in the cross-national literature. Using 2-way fixed and generalized least squares random effects models for a sample of 74 low- and middle-income nations from 1996 to 2012, I examine how the interaction among 5 measures of national governance and public medical care expenditures impact child mortality. The findings reveal the importance of governance in determining the effectiveness of public medical care expenditures. Both public medical care expenditures and governance improvements are essential to reduce child mortality.

Over the past 3 decades, we have seen vast improvements in health and well-being across most nations.^1^ This accomplishment is in part due to nations prioritizing and increasing health spending.^2–4^ Despite these achievements, approximately 6.3 million children under the age of 5 die annually.^1^ Moreover, child mortality rates are not distributed equally. In 2015, child deaths per 1,000 live births in low- and middle-income nations were approximately 6 times greater than these deaths in high-income nations.^1^ How can we further improve the capacity for well-being in developing nations?

Researchers argue that continuing to expand government services and funding for health should further decrease child mortality.^2,4,5^ Public medical care expenditures can reduce child mortality because they fund resources to improve health care facilities, which provide immunizations, family planning, and childbirth care.^6–8^ For example, in a cross-national study, Filmer and Pritchett^9^ find that increases in public health spending were associated with reduced child mortality.

In contrast, other researchers argue that public medical care expenditures may not improve child health if a state does not allocate and implement funds to areas in most need.^10–12^ In some nations, for example, there are not enough funds for health spending to support the entire population, or resources are not available to citizens in remote regions and rural areas.^7,9,13,14^ Therefore, some nations’ public spending could be ineffective at reducing child mortality. For instance, Shandra et al.^15^ find an inconsistent cross-national relationship, and Pandolfelli and Shandra^16^ find no cross-national relationship, between public medical care expenditures and child mortality.

Therefore, increasing public medical care expenditures might not yield the desired outcomes. Building on this debate, I argue that governance may be the moderating factor dividing these 2 research perspectives. Low levels of governance can prevent the state from delivering and redistributing health services for its citizens.^17^ Yet, few cross-national and longitudinal studies examine how governance is associated with the effectiveness of public medical care expenditures. Extant studies on public medical care expenditures and child health have found mixed results. Rajkumar and Swaroop^10^ find that corruption and bureaucracy interact with public medical care expenditures to impact child mortality rates. However, Hu and Mendoza find no support for an interaction between governance, measured through bureaucracy and control of corruption, and public medical care expenditures on child health outcomes.^18^

Overall, studies find inconsistent relationships between public medical care expenditures and child mortality.^15,16,19–24^ This article builds on previous research by interacting 5 different measures of governance using the World Bank’s World Governance Indicators (which include measures for rule of law, regulatory quality, government effectiveness, political stability, and control of corruption) with nations’ public medical care expenditures in the most recent time period to assess whether governance and public medical care expenditures are effective at decreasing child mortality.^25^

Using 2-way fixed effects and generalized least squares random effects regression models for a sample of 74 low- and middle-income nations from 1996–2012, I test the claim that health expenditures reduce child mortality more in nations with higher levels of governance rather than lower levels of governance. Thus, I argue that proper allocation, effectiveness, and the quality of health services are essential for health expenditures to improve citizen health. Below, I discuss the theoretical relationship between public medical care expenditures and child mortality. After, I describe how factors of governance may interact with public health spending and what the implications are for child health. Finally, I discuss the variables, methodology, findings, and conclusion.

## Government Spending on Health

Nations’ characteristics explain differences in health and well-being in their populations because states make internal decisions, such as how resources are distributed among different sectors or what policies and practices they enforce.^6,24,26–28^ Government health resources, in particular, are considered essential for improving health in developing nations.^8,29,30^

Nations often use public medical care expenditures to invest in large-scale health programs that include personal training, hospital updates, equipment, and primary care.^1,7,8,31^ Public medical care expenditures fund everything from immunizations to family planning.^1,7^ During pregnancy and after birth, public health spending provides prenatal care, postnatal care, and nutrition counseling for mothers and their children.^1,7^ Public health spending also aims to increase the social development and health of a nation’s population. Unfortunately, mothers and their children are often the most vulnerable to failed health spending, because this population is most at risk for easily preventable diseases and illnesses.^9,16,32^

If a nation’s health spending does not reach its intended destination in full, or if funds are used for other purposes, these activities may not improve child mortality.^7^ In short, health spending must be allocated to relevant causes and areas in most need in developing nations to effectively improve child health.^13,33^ The ability of the state to effectively allocate health resources can be measured by a nation’s governance. Governance is one potential solution to the ineffectiveness of public health spending because it measures how well a nation can redistribute resources, ensure resources reach their intended goal, and confirm resources go toward useful ends.

## Governance: Accountable Government Health Spending

Although states must have the resources to allocate enough funds to cover the health needs of their populations, how resources are utilized depends on what structures are in place and how the government is characterized.^10,26^ A nation must have adequate channels and avenues for the successful delivery of health services, as well as proper planning and implementation to ensure funds are used as efficiently for the target population as possible. Although many researchers argue that public medical care expenditures increase child health, others find these efforts ineffective.^19^ Building on this previous research concerning the effectiveness of health spending, I argue that although governments expend large amounts of funds for public health to improve outcomes in developing nations, expenditures in and of themselves are necessary, but not sufficient to improve child health. Directing expenditures so that they are most effective and efficient is crucial.^16,34^ In particular, I claim that governance is an important moderating factor that provides the missing element in these 2 bodies of research. I fill this lacuna by testing how 5 types of governance interact with public medical care expenditures.

According to multiple World Bank studies by Kaufmann et al.^35^ and Kaufmann and Kraay,^25^ governance is classified into 5 dimensions. These include political stability, control of corruption, rule of law, government effectiveness, and regulatory quality. Below I describe how these 5 measures of governance should interact with public medical care expenditures to provide an integrated theory of accountable government spending and well-being. A summary of how each aspect of governance can improve the effectiveness of public medical care expenditures is below in Table 1.

**Table 1.** The Relationships Between Government Health Spending and Good Governance.

Governance Measures	Relationship to Public Medical Care Expenditures
Political stability	Encourages budgetary management, ensuring that all sectors get equitable attention (i.e., health sectors) and increases service delivery
Control of corruption	Controlling corruption in a state can limit leakage of health resources
Rule of law	Enforces intention of funds, increases contract enforcement, and encourages distribution
Government effectiveness	Encourages coherent strategies to fund relevant activities to population
Regulatory quality	Regulatory quality can create mutual partnerships with private sector to create incentive programs for health

First, political stability reflects the level of political conflict and violence within a nation.^25^ Health care facilities are often flooded with injured victims in conflict-stricken nations. Unfortunately, this limits the amount of funds and care available for children.^36–38^ For example, Ghobarah et al.^36^ find that civil war is responsible for poor citizen health even after conflict is ceased, especially in children. However, politically stable nations do not suffer from these problems. Government funds to settle conflicts may detract from funds available for health, leaving parents and their children without necessary care.^39^ Moreover, political instability may result in poor budgeting and unequal distribution of funds for health and may complicate the delivery of health resources.^7,9^ In contrast, politically stable nations can ensure all sectors get equitable attention and have more reliable service delivery.^37,38^

Second, corruption reduces the amount of public medical care expenditures available,^39^ which can leave health facilities without the equipment and staff necessary to provide efficient and effective care.^40^ When fewer health funds are available as a result of corruption, health facilities often collect unofficial fees from patients to stay open.^13^ Unfortunately, citizens may be unable to afford these unofficial fees or bribes for care and materials, which leads to more children dying from treatable illnesses.^14,41^ For example, Parsitau^14^ finds that in Kenya, user fees dissuade women from attending doctor visits for prenatal care. When women cannot afford to purchase sanitary materials (such as gloves) for childbirth, they are at a higher risk for infections and pregnancy complications that can cause death. However, if a state can control corruption, the misappropriation of health resources is limited, allowing funds and materials to reach their destination.^42,43^

Third, rule of law concerns the ability of a state to enforce the rights of citizens within its borders.^20,25^ The combination of rule of law and public medical care expenditures improves the enforcement of the purpose of health funds.^24,25^ In particular, states that have command over their full territory have the ability to establish clear pathways for the distribution of health spending, resulting in more consistent dispersion of funds and materials necessary to reduce child mortality.^16,24,44^ However, nations with low levels of rule of law will not have the institutional structures necessary to disseminate health funds to populations in remote areas that are most at risk for poor health outcomes.^44,45^

Fourth, government effectiveness concerns the quality of public and civil government services and policies.^25^ Government effectiveness should increase the efficacy of public spending by encouraging coherent and relevant plans for public medical care expenditures.^25,46,47^ Therefore, government effectiveness can help increase the relevance and proper allocation of health funds.^46,47^ However, government inability to use funds properly through low government effectiveness leads to decreased health outcomes.^33^ According to Filmer and Pritchett,^9^ public medical care expenditures in India are frequently allocated to expensive medical technologies in hospitals intended to treat the rich, while children die from diseases that are cheap to prevent and cure.

Fifth, regulatory quality is defined as the ability of the government to establish mutually beneficial relationships with the private sector.^25^ Nations with high levels of regulatory quality establish agreements with the private sector to create greater health care access for their population by increasing fund availability and assisting with service delivery.^48^ Moreover, effective governments pressure their private sector to invest in health by providing incentives to those that make efforts to improve health outcomes.^10,48^ In contrast, nations with low regulatory quality may not have sufficient control over private-sector development, which can result in competition between private and public facilities, reducing the quality of health services and the effectiveness of public health spending.^19,25^

Institutional structures combined with government spending (outlined in Table 1) should enable nations to promote effective and efficient public health spending, increase the amount of health funds available, enforce the intention of and prioritize those funds, follow coherent strategies for the best use of funds, and create transparent partnerships with the private sector to generate more resources for public medical care expenditures. Effective allocation of health spending depends on a state’s ability to implement these ideals.^10,18^ Below I discuss the methodology and data used in this study.

## Methods

### Sample

Following previous studies, the present research focuses on low- and middle-income nations because they have lower levels of health expenditures and governance and higher levels of child mortality compared to high-income nations.^1,24,49^ Listwise deletion of missing data yields an unbalanced panel of 74 country years (1996–2012), including 634 observations, with a minimum of 2, an average of 8, and a maximum of 16 years per country. Table 2 includes descriptive statistics and bivariate correlation matrix. The sample can be found in Supplementary Materials.

**Table 2.** Descriptive Statistics and Bivariate Correlation Matrix.

Variable											Mean			SD					Min		Max
Child mortality											3.875			0.807					1.723		5.442
Health expenditure, public (% of GDP)											2.685			1.237					0.252		7.613
Political stability											−0.472			0.802					−2.812		1.308
Control of corruption											−0.490			.516					−1.640		1.140
Rule of law											−0.505			0.532					−1.947		0.991
Government effectiveness											−0.409			0.548					−1.742		1.247
Regulatory quality											−0.350			0.533					−1.851		0.898
Tax revenue (% of GDP) (ln)											2.615			0.478					−1.417		4.111
GDP (per capita) (ln)											7.064			1.007					4.702		9.216
GDP growth											5.022			4.042					−14.800		25.049
Trade (% of GDP)											83.668			38.853					21.552		220.407
Multinational corporate investment (% of GDP) (ln)											3.022			0.954					.000		6.321
Domestic investment (ln)											3.131			0.332					1.518		4.374
Total population											46,400,000			149,000,000					147,455		1,170,000,000
Measles immunizations											82.484			15.224					34.000		99.000
Human immunodeficiency virus prevalence (ln)											−0.419			1.604					−2.303		3.246
Female education											47.578			5.579					23.552		61.041
Democracy											12.268			7.709					0.000		34.900
Access to improved water/sanitation											67.502			21.144					12.200		97.650
(1)		(2)		(3)		(4)		(5)		(6)			(7)			(8)		(9)		(10)	
1.000																					
−0.209		1.000																			
−0.182		0.499		1.000																	
−0.261		0.419		0.570		1.000															
−0.267		0.269		0.581		0.843		1.000													
−0.416		0.204		0.418		0.803		0.833		1.000											
−0.350		0.171		0.351		0.612		0.672		0.762			1.000								
−0.157		0.273		0.335		0.272		0.310		0.310			0.322			1.000					
−0.710		0.217		0.310		0.402		0.340		0.509			0.476			0.359		1.000			
0.065		−0.100		−0.010		−0.082		−0.075		−0.074			−0.174			−0.121		−0.064		1.000	
−0.304		0.198		0.301		0.112		0.134		0.145			0.018			0.284		0.217		0.035	
−0.211		0.119		0.197		0.044		0.076		0.082			0.222			0.209		0.351		−0.079	
−0.193		0.195		0.262		0.279		0.317		0.236			0.012			0.100		0.067		0.161	
0.096		−0.275		−0.241		−0.031		0.130		0.081			−0.019			−0.156		−0.104		0.060	
−0.667		0.304		0.323		0.260		0.282		0.327			0.324			0.196		0.494		−0.055	
0.439		0.206		0.153		0.131		0.069		−0.022			0.118			0.282		−0.119		−0.095	
−0.558		0.301		0.291		0.356		0.357		0.432			0.352			0.367		0.510		−0.093	
−0.473		0.076		0.004		0.028		0.112		0.110			0.275			0.028		0.242		−0.088	
−0.863		0.158		0.150		0.226		0.237		0.419			0.326			0.200		0.723		−0.076	
(11)	(12)		(13)		(14)		(15)		(16)			(17)			(18)		(19)				
1.000																					
0.403	1.000																				
0.136	−0.036		1.000																		
−0.251	−0.238		0.078		1.000																
0.248	0.158		0.194		−0.209		1.000														
0.066	0.190		−0.208		−0.104		−0.347		1.000												
0.312	0.241		0.279		−0.152		0.546		−0.088			1.000									
−0.011	−0.004		0.037		0.201		0.306		−0.176			0.306			1.000						
0.257	0.220		0.108		−0.077		0.682		−0.451			0.583			0.344		1.000				

Abbreviation: GDP, gross domestic product.

### Statistical Models

Due to the availability of panel data, this study uses estimation techniques that correct for heterogeneity bias.^50,51^ This study uses 2-way fixed effects and generalized least squares random effects regression models with robust standard errors clustered by country to examine the effect of public medical care expenditures and governance on child mortality. According to the Sargen-Hansen test in Tables 3 and 4, 2-way fixed effects may be more appropriate than generalized least squares random effects.However, the Hausman test in Tables 3 and 4 reveals mixed results. Therefore, I report both 2-way fixed effects and generalized least squared random effects. Similar findings across both model types enhance the reliability of the results. The models include time dummy variables for each year (1996–2012). In addition, several post estimation tests reveal no issues with outliers and multicollinearity.^52^ Furthermore, I take the natural logarithm of variables when they are skewed and note it in Table 2. More information on data, variable, and model selection as well as regression assumptions are in the Supplementary Files. I use a 1-tailed test of statistical significance due to the directional nature of the hypothesis.

**Table 3.** Two-Way Fixed Effects Estimates of Public Health Spending, Governance, and Child Mortality, 1996–2012.

Independent Variables	(3.1)	(3.2)	(3.3)	(3.4)	(3.5)
Health expenditure, public (% of GDP)	0.022	0.022	0.022	0.021	0.022
	0.034	0.034	0.034	0.033	0.034
	(0.014)	(0.014)	(0.014)	(0.014)	(0.014)
Political stability	0.028				
	0.028				
	(0.021)				
Control of corruption		0.007			
		0.005			
		(0.032)			
Rule of law			0.036		
			0.024		
			(0.041)		
Government effectiveness				0.031	
				0.021	
				(0.041)	
Regulatory quality					0.023
					0.015
					(0.031)
Tax revenue (% of GDP)	−0.021	−0.019	−0.023	−0.023	−0.020
	−0.012	−0.011	−0.015	−0.013	−0.012
	(0.039)	(0.039)	(0.041)	(0.039)	(0.040)
GDP (per capita)	−0.133***	−0.118**	−0.124**	−0.122**	−0.124***
	−0.166	−0.147	−0.155	−0.152	−0.155
	(0.039)	(0.043)	(0.042)	(0.042)	(0.041)
GDP growth	0.001	0.001	0.001	0.001	0.001
	0.006	0.006	0.006	0.006	0.006
	(0.001)	(0.001)	(0.001)	(0.001)	(0.001)
Total population	0.001***	0.001***	0.001***	0.001***	0.001***
	0.286	0.272	0.277	0.259	0.266
	(0.001)	(0.001)	(0.001)	(0.001)	(0.001)
Measles immunizations	0.001	0.001	0.001	0.001	0.001
	0.015	0.018	0.015	0.016	0.017
	(0.001)	(0.001)	(0.001)	(0.001)	(0.001)
Human immunodeficiency virus prevalence	0.004	0.003	0.003	0.005	0.001
	0.007	0.007	0.006	0.011	0.001
	(0.045)	(0.046)	(0.046)	(0.044)	(0.046)
Trade (% of GDP)	−0.002**	−0.002**	−0.002**	−0.002**	−0.002**
	−0.083	−0.082	−0.084	−0.081	−0.083
	(0.001)	(0.001)	(0.001)	(0.001)	(0.001)
Female education	−0.009*	−0.009*	−0.009*	−0.009*	−0.009*
	−0.062	−0.062	−0.062	−0.059	−0.061
	(0.004)	(0.004)	(0.004)	(0.004)	(0.004)
Multinational corporate investment (% of GDP)	−0.048**	−0.049**	−0.050**	−0.050**	−0.050**
	−0.056	−0.059	−0.059	−0.059	−0.059
	(0.017)	(0.018)	(0.018)	(0.018)	(0.018)
Domestic investment	−0.089*	−0.086*	−0.085*	−0.086*	−0.087*
	−0.037	−0.036	−0.035	−0.035	−0.036
	(0.046)	(0.045)	(0.047)	(0.046)	(0.046)
Democracy	−0.002	−0.001	−0.001	−0.001	−0.001
	−0.015	−0.013	−0.013	−0.013	−0.013
	(0.001)	(0.001)	(0.001)	(0.001)	(0.001)
Access to improved water/sanitation	−0.010*	−0.010*	−0.010*	−0.010*	−0.010*
	−0.261	−0.269	−0.264	−0.270	−0.267
	(0.006)	(0.006)	(0.006)	(0.006)	(0.006)
Overall R-Square	0.553	0.555	0.545	0.562	0.554
Within R-Square	0.882	0.880	0.881	0.881	0.881
Number of observations	634	634	634	634	634
Number of countries	74	74	74	74	74
Sargan-Hansen Test statistic	57.748***	53.358***	53.115***	51.436***	53.642***
Hausman Test	10.25	0.59	18.99	23.62	23.12

Abbreviation: GDP, gross domestic product.

The first number is the unstandardized coefficient, the second is the standardized beta, and the robust standard error is in parentheses. The null hypothesis for the Sargan-Hansen test is that the random effects estimator is more efficient than the fixed effects estimator. The null hypothesis for the Hausman test is that the difference in coefficients is not systematic.

*P < .05; **P < .01; ***P < .001 for a 1-tailed test.

**Table 4.** Two-Way Fixed Effects Estimates of Public Health Spending, Governance, and Child Mortality, 1996–2012.

Independent Variables	(4.1)	(4.2)	(4.3)	(4.4)	(4.5)
Political stability × Health expenditure, public	−0.022*				
	−0.053				
	(0.011)				
Control of corruption × Health expenditure, public		−0.048**			
		−0.092			
		(0.017)			
Rule of law × Health expenditure, public			−0.036*		
			−0.066		
			(0.018)		
Government effectiveness × Health expenditure, public				−0.034**	
				−0.067	
				(0.014)	
Regulatory quality × Health expenditure, public					−0.062***
					−0.124
					(0.020)
Health expenditure, public (% of GDP)	0.018	0.003	0.005	0.005	0.008
	0.028	0.005	0.008	0.008	0.012
	(0.013)	(0.015)	(0.017)	(0.015)	(0.015)
Political stability	0.082				
	0.081				
	(0.040)				
Control of corruption		0.142			
		−0.091			
		(0.051)			
					
Rule of law			0.129		
			−0.085		
			(0.068)		
Government effectiveness				0.128	
				0.087	
				(0.056)	
Regulatory quality					0.177
					0.117
					(0.058)
Tax revenue (% of GDP)	−0.032	−0.027	−0.036	−0.027	−0.039
	−0.019	−0.016	−0.022	−0.016	−0.023
	(0.038)	(0.036)	(0.040)	(0.038)	(0.036)
GDP (per capita)	−0.142***	−0.132***	−0.133***	−0.132***	−0.131***
	−0.177	−0.165	−0.166	−0.165	−0.163
	(0.039)	(0.041)	(0.041)	(0.041)	(0.039)
GDP growth	0.001	0.001	0.001	0.001	0.001
	0.005	0.006	0.005	0.006	0.004
	(0.001)	(0.001)	(0.001)	(0.001)	(0.001)
Total population	0.001***	0.001***	0.001***	0.001***	0.001***
	0.306	0.291	0.294	0.270	0.266
	(0.001)	(0.001)	(0.001)	(0.001)	(0.001)
Measles immunizations	0.001	0.001	0.001	0.001	0.001
	0.013	0.014	0.010	0.011	0.011
	(0.001)	(0.001)	(0.001)	(0.001)	(0.001)
Human Immunodeficiency Virus prevalence	0.003	−0.006	0.001	−0.001	−0.007
	0.007	−0.013	0.001	−0.003	−0.013
	(0.045)	(0.045)	(0.045)	(0.044)	(0.042)
Trade (% of GDP)	−0.002***	−0.002***	−0.002***	−0.002***	−0.002***
	−0.087	−0.088	−0.089	−0.087	−0.086
	(0.001)	(0.001)	(0.001)	(0.001)	(0.001)
Female education	−0.009**	−0.009**	−0.009**	−0.009**	−0.008*
	−0.064	−0.063	−0.064	−0.063	−0.055
	(0.004)	(0.004)	(0.004)	(0.004)	(0.004)
Multinational corporate investment (% of GDP)	−0.050**	−0.055***	−0.052**	−0.052**	−0.049**
	−0.060	−0.066	−0.060	−0.062	−0.057
	(0.017)	(0.018)	(0.018)	(0.018)	(0.019)
Domestic investment	−0.086*	−0.095*	−0.084*	−0.088*	−0.083*
	−0.035	−0.039	−0.035	−0.036	−0.034
	(0.046)	(0.047)	(0.049)	(0.047)	(0.044)
Democracy	−0.002	−0.001	−0.002	−0.001	−0.001
	−0.020	−0.012	−0.015	−0.013	−0.011
	(0.001)	(0.001)	(0.001)	(0.001)	(0.001)
Access to improved water/sanitation	−0.010*	−0.010*	−0.010*	−0.010*	−0.012**
	−0.274	−0.275	−0.261	−0.271	−0.323
	(0.006)	(0.005)	(0.006)	(0.006)	(0.005)
Overall R-Square	0.553	0.519	0.511	0.535	0.561
Within R-Square	0.884	0.888	0.884	0.885	0.891
Number of observations	634	634	634	634	634
Number of countries	74	74	74	74	74
Sargan-Hansen Test statistic	88.649***	58.592***	69.468***	62.063***	57.729***
Hausman Test	45.18	48.27*	57.24*	45.15*	23.48

Abbreviation: GDP, gross domestic product.

The first number is the unstandardized coefficient, the second is the standardized beta, and the robust standard error is in parentheses. The null hypothesis for the Sargan-Hansen test is that the random effects estimator is more efficient than the fixed effects estimator. The null hypothesis for the Hausman test is that the difference in coefficients is not systematic.

*P < .05; **P < .01; ***P < .001 for a 1-tailed test.

### Two-Way Fixed Effects


$$y_{\text{it}}=a+B_{\text{1}}x_{\text{it1}}+B_{\text{2}}x_{\text{it2}}+\cdot \cdot \cdot \cdot +B_{\text{k}}x_{\text{itk}}+u_{\text{i}}+w_{\text{t}}+e_{\text{it}}$$

where *i* = each country in the analysis, *t* = each time period in the analysis, *y*_it_ = dependent variable for each country at each time period, *a* = the constant, *B_1_ to B*_k_ = coefficients for each independent variables,*x*_itk_ = independent variables for each country at each time point, *u*_i_ = country-specific disturbance terms that are constant over time, *w*_t_ = period-specific disturbance terms that are constant across all countries,and *e*_it_ = disturbance terms specific to each country at each time point.

## Data

### Dependent Variable

#### Child Mortality

The dependent variable measures the probability per 1,000 live births that a newborn baby will die before age 5.^1^ This measure is logged due to skewness. Please note that all data are publicly available from the World Bank^1^ unless directly indicated in the following sections.

### Main Independent Variables

#### Health Expenditure, Public (% of Gross Domestic Product [GDP])

Public medical care expenditures reflect current health spending for medical services by every level of a nation’s government.^53^ According to the World Bank,^1^

Public health expenditure consists of recurrent and capital spending from government (central and local) budgets, external borrowings and grants (including donations from international agencies and nongovernmental organizations), and social (or compulsory) health insurance funds.

As detailed above, higher levels of public medical care expenditures should relate to lower levels of child mortality.

#### Governance

I use data from the World Governance Indicators database by the World Bank to measure political stability, control of corruption, rule of law, government effectiveness, and regulatory quality (see www.govindicators.org for more details on the creation and aggregation of these data).^25^ These data range from −2.5 to 2.5, where a score of −2.5 represents very low governance and a score of 2.5 represents very high governance. These newly available data are indices based on several cross-national surveys and insights from experts, nongovernmental organizations, and research bodies.^35^ Although these data are similar to measurements of governance from Transparency International and the International Country Risk Guide Index, they provide more country-years and disaggregation among multi-dimensional aspects of governance. Therefore, these data are a great improvement on previous measures in terms of cross-national coverage and theoretical relevance.^35^ Due to data availability, the Worldwide Governance Indicators data did not include the years 1997, 1999, and 2001. To include these years, I lagged and led the data by averaging years surrounding the years not available (i.e., I averaged 2000 and 2002 to get 2001).^1^

### Control Variables

Based on previous research on child mortality, I include several control variables.^24,53^ The economic control variables are *tax revenue,*^4^ *GDP per capita*, *GDP growth*, *multinational corporate investment*,^54,55^ *domestic investment*,^56^ and *trade.*^28^ I also include a control for *democracy* from Vanhanen’s competition and political participation index.^57–59^ Last, I include a number of social controls, including *secondary school enrollment % pupils female,*^9^ *human immunodeficiency virus prevalence*,^60,61^ *measles immunizations*,^60^ an index for access to *improved water and sanitation*,^56^ and *total population.*^62^ Other control variables were considered and later removed for issues with sample size reduction, overspecification, theoretical relevance, and non-significance.

## Findings

Table 3 uses 2-way fixed effects regression and Table 5 uses generalized least squares random effects regression to examine the linear effects of public medical care expenditures and governance on child mortality. Table 4 uses 2-way fixed effects regression and Table 6 uses generalized least squares random effects regression to test the interactive effects of public medical care expenditures and each governance measure. Because the measures of governance are highly correlated with each other, each equation contains 1 of the 5 measures of governance. Further, these variables measure different aspects of governance and may have differing effects on child mortality.^63^

**Table 5.** Generalized Least Squares Random Effects Estimates of Public Health Spending, Governance, and Child Mortality, 1996–2012.

	(3.1)	(3.2)	(3.3)	(3.4)	(3.5)
Independent Variables					
Health expenditure, public (% of GDP)	0.019	0.020	0.020	0.019	0.020
	0.030	0.030	0.030	0.029	0.030
	(0.014)	(0.014)	(0.014)	(0.014)	(0.014)
Political stability	0.028				
	0.028				
	(0.021)				
Control of corruption		0.002			
		0.001			
		(0.032)			
Rule of law			0.022		
			0.014		
			(0.042)		
Government effectiveness				0.028	
				0.019	
				(0.045)	
Regulatory quality					0.014
					0.009
					(0.028)
Tax revenue (% of GDP)	−0.029	−0.026	−0.029	−0.031	−0.028
	−0.017	−0.016	−0.017	−0.018	−0.016
	(0.035)	(0.035)	(0.037)	(0.035)	(0.037)
GDP (per capita)	−0.160***	−0.144**	−0.148***	−0.149***	−0.149***
	−0.199	−0.180	−0.185	−0.186	−0.186
	(0.041)	(0.045)	(0.046)	(0.046)	(0.045)
GDP growth	0.002*	0.002*	0.002*	0.002*	0.002*
	0.008	0.008	0.009	0.009	0.009
	(0.001)	(0.001)	(0.001)	(0.001)	(0.001)
Total population	0.001***	0.001***	0.001***	0.001***	0.001***
	0.153	0.143	0.143	0.135	0.140
	(0.001)	(0.001)	(0.001)	(0.001)	(0.001)
Measles immunizations	0.001	0.001	0.001	0.001	0.001
	0.022	0.026	0.024	0.024	0.025
	(0.001)	(0.001)	(0.001)	0.001	(0.001)
Human immunodeficiency virus prevalence	0.027	0.028	0.027	0.029	0.027
	0.054	0.055	0.055	0.058	0.053
	(0.037)	(0.038)	(0.038)	(0.036)	(0.037)
Trade (% of GDP)	−0.002**	−0.002***	−0.002**	−0.002**	−0.002**
	−0.090	−0.089	−0.090	−0.089	−0.090
	(0.001)	(0.001)	(0.001)	(0.001)	(0.001)
Female education	−0.008*	−0.008*	−0.008*	−0.008*	−0.008*
	−0.056	−0.056	−0.056	−0.054	−0.055
	(0.004)	(0.004)	(0.004)	(0.004)	(0.004)
Multinational corporate investment (% of GDP)	−0.050***	−0.052***	−0.053**	−0.053***	−0.052**
	−0.060	−0.061	−0.062	−0.062	−0.062
	(0.016)	(0.017)	(0.018)	(0.045)	(0.018)
Domestic investment	−0.091*	−0.089*	−0.088*	−0.088*	−0.089*
	−0.037	−0.037	−0.036	−0.036	−0.037
	(0.045)	(0.044)	(0.045)	(0.045)	(0.045)
Democracy	−0.002	−0.002	−0.002	−0.002	−0.001
	−0.022	−0.020	−0.020	−0.020	−0.021
	(0.001)	(0.001)	(0.001)	(0.001)	(0.001)
Access to improved water/sanitation	−0.016***	−0.017***	−0.017***	−0.017***	−0.017***
	−0.428	−0.439	−0.436	−0.444	−0.440
	(0.006)	(0.004)	(0.004)	(0.003)	(0.004)
Constant	7.217***	7.108***	7.155****	7.170***	7.146***
	(0.276)	(0.298)	(0.294)	(0.299)	(0.480)
Overall R-Square	0.737	0.738	0.736	0.740	0.738
Between R-Square	0.770	0.771	0.768	0.771	0.770
Number of observations	634	634	634	634	634
Number of countries	74	74	74	74	74

Abbreviation: GDP, gross domestic product.

The first number is the unstandardized coefficient, the second is the standardized beta, and the robust standard error is in parentheses.

*P < .05; **P < .01; ***P < .001 for a 1-tailed test.

**Table 6.** Generalized Least Squares Random Effects Estimates of Public Health Spending, Governance, and Child Mortality, 1996–2012.

	(4.1)	(4.2)	(4.3)	(4.4)	(4.5)
Independent Variables					
Political stability × Public medical care expenditures	−0.022*				
	−0.055				
	(0.012)				
Control of corruption × Public medical care expenditures		−0.044**			
		−0.083			
		(0.051)			
Rule of law × Public medical care expenditures			−0.029		
			−0.052		
			(0.019)		
Government effectiveness × Public medical care expenditures				−0.030*	
				−0.059	
				(0.014)	
Regulatory quality × Public medical care expenditures					−0.063***
					−0.126
					(0.020)
Public medical care expenditures (% of GDP)	0.016	0.003	0.006	0.005	−0.010
	0.024	0.005	0.010	0.007	−0.015
	(0.013)	(0.014)	(0.016)	(0.015)	(.014)
Political stability	0.083*				
	0.083				
	(0.042)				
Control of corruption		0.125**			
		00.080			
		(0.051)			
Rule of law			0.094		
			0.062		
			(0.068)		
Government effectiveness				0.115*	
				0.037	
				(0.055)	
Regulatory quality					0.172**
					0.114
					(0.058)
Tax revenue (% of GDP)	−0.039	−0.034	−0.023	−0.035	−0.046
	−0.023	−0.020	−0.022	−0.021	−0.027
	(0.034)	(00.033)	(0.036)	(0.034)	(0.033)
GDP (per capita)	−0.166***	−0.155***	−0.155***	−0.158***	−0.150***
	−0.208	−0.193	−0.193	−0.197	−0.188
	(0.042)	(0.044)	(0.045)	(0.045)	(0.040)
GDP growth	0.002*	0.002*	0.002*	0.002*	0.001
	0.008	0.008	0.008	0.008	0.006
	(0.001)	(0.001)	(0.001)	(0.001)	(0.001)
Total population	0.001***	0.001***	0.001***	0.001***	0.001***
	0.162	0.151	0.142	0.131	0.136
	(0.001)	(0.001)	(0.001)	(0.001)	(0.001)
Measles immunizations	0.001	0.001	0.001	0.001	0.001
	0.019	0.021	0.020	0.019	0.017
	(0.001)	(0.001)	(0.001)	(0.001)	(0.001)
Human immunodeficiency virus prevalence	0.027	0.021	0.027	0.026	0.020
	0.054	0.075	0.054	0.051	0.040
	(0.037)	(0.038)	(0.037)	(0.036)	(0.036)
Trade (% of GDP)	−0.002***	−0.002***	−0.002***	−0.002**	−0.002***
	−0.093	−0.093	−0.093	−0.093	−0.091
	(0.001)	(0.001)	(0.001)	(0.001)	(0.001)
Female education	−0.008**	−0.008**	−0.089*	−0.008**	−0.007*
	−0.057	−0.055	−0.053	−0.055	−0.048
	(0.003)	(0.003)	(0.003)	(0.003)	(0.003)
Multinational corporate investment (as a % of GDP)	−0.053***	−0.057***	−0.054***	−0.055***	−0.050**
	−0.062	−0.067	−0.064	−0.064	−0.059
	(0.016)	(0.018)	(0.017)	(0.018)	(0.018)
Domestic investment	−0.088*	−0.097*	−0.088*	−0.090*	−0.086*
	−0.036	−0.040	−0.036	−0.037	−0.035
	(0.045)	(0.045)	(0.046)	(0.045)	(0.042)
Democracy	−0.003*	−0.002	−0.002	−0.002	−0.002
	−0.027	−0.019	−0.022	−0.021	−0.018
	(0.001)	(0.001)	(0.001)	(0.001)	(0.001)
Access to improved water/sanitation	−0.016***	−0.016***	−0.017***	−0.017***	−0.018***
	−0.427	−0.431	−0.433	−0.440	−0.459
	(0.004)	(0.003)	(0.004)	(0.003)	(0.003)
Constant	7.332***	7.294***	7.268***	7.325***	7.312***
	(0.281)	(0.302)	(0.301)	(0.289)	(0.280)
Overall R-Square	0.731	0.714	0.724	0.729	0.720
Between R-Square	0.764	0.752	0.759	0.763	0.757
Number of observations	634	634	634	634	634
Number of countries	74	74	74	74	74

Abbreviation: GDP, gross domestic product.

The first number is the unstandardized coefficient, the second is the standardized beta, and the robust standard error is in parentheses.

*P < .05; **P < .01; ***P < .001 for a 1-tailed test.

Tables 3 and 5 show that the coefficients that represent public medical care expenditures and all 5 governance measures fail to reach levels of statistical significance. This diverges from research by Hu and Mendoza.^18^ However, a number of other factors are related to child mortality. First, a number of macroeconomic variables are associated with less child mortality. The coefficients that represent GDP per capita, trade, foreign direct investment, and domestic investment are negative and significant in every equation. Second, the coefficients that represent total population are positive and significant, which suggests that higher levels of total population are associated with increased child mortality. Third, the coefficients that represent access to female education and water and sanitation are negative and significant in every equation. This suggests that higher levels of access to female education and water and sanitation correspond with lower levels of child mortality. In the random effects models in Table 5, the coefficients that represent GDP growth are positive and statistically significant but fail to reach levels of statistical significance in the fixed effects models in Table 3. This may be due to a lack of variation of GDP growth from year to year, but large variation from nation to nation.

In Table 4, the coefficients that represent each interaction term^6^ are negative and significant in every equation (except for the interaction between rule of law and public medical care expenditures in the random effects model in Table 6). The sign and significance of these coefficients generally suggest that public medical care expenditures decrease child mortality more at higher levels than at lower levels of state governance.

**Figure 1.**
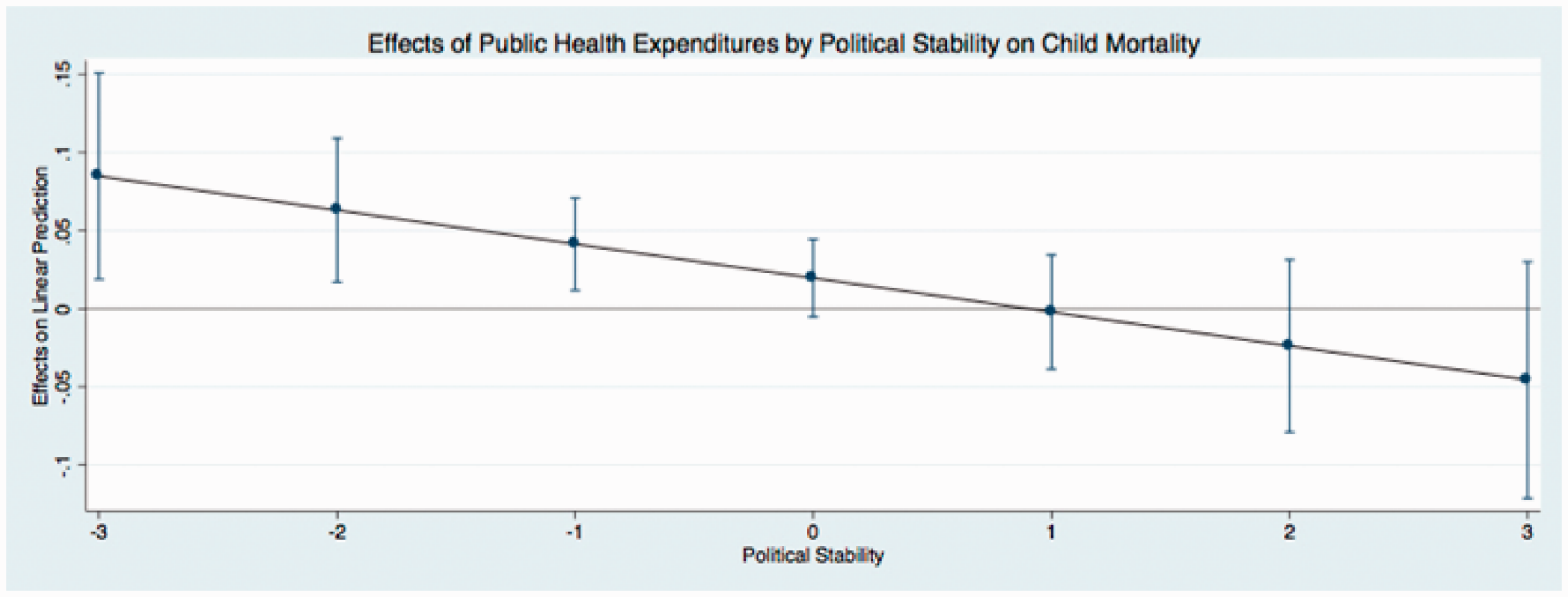
Predicted effects: effects of public health expenditures by political stability on child mortality.

The predicted effects of these relationships (see Figure 1) illustrate that public expenditures have different effects on child mortality at different levels of governance. In these figures, I use the coefficients from Table 4 to graph the predicted change in public medical care expenditures as governance simultaneously increases, holding all continuous covariates at their mean and categorical covariates (time dummy variables) at the reference category of zero. I find that the effect of public medical care expenditures on child mortality is relatively low when governance is low. This indicates that when public medical care expenditures and governance are low, there are higher levels of child mortality. Initial increases in governance results in an incline in public medical care expenditures, supporting the hypotheses of this study. In particular, as public medical care expenditures and governance increase, child mortality steadily declines (as indicated by the downward sloping line). The other findings mostly remain stable and consistent across the new model specifications, with the exception of the coefficients that represent democracy in 2 equations in Table 6. These results are similar to the results reported in Table 3.

**Figure 2.**
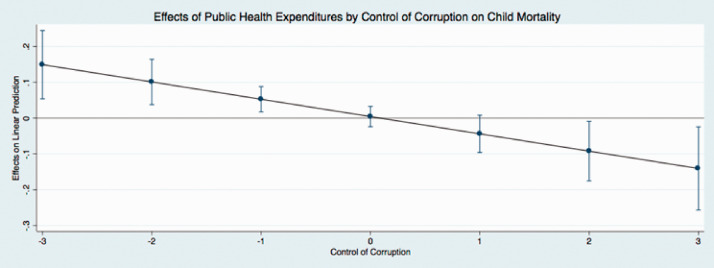
Predicted effects: effects of public health expenditures by control of corruption on child mortality.

**Figure 3.**
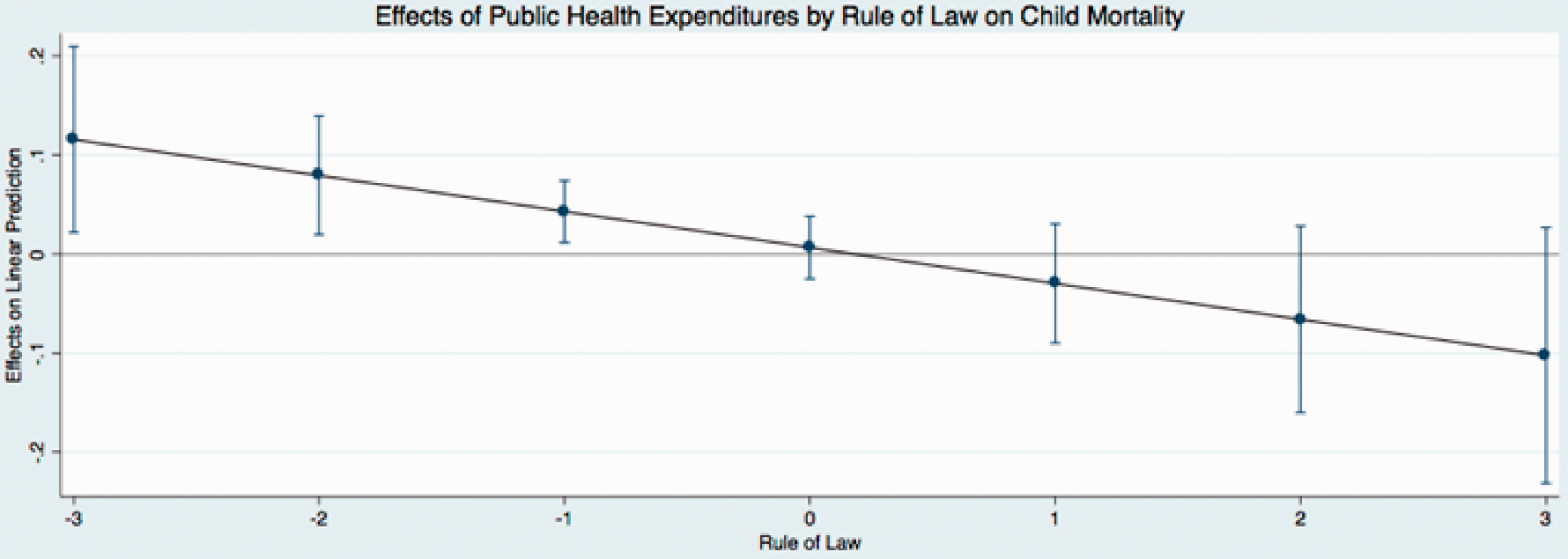
Predicted effects: effects of public health expenditures by rule of law on child mortality.

**Figure 4.**
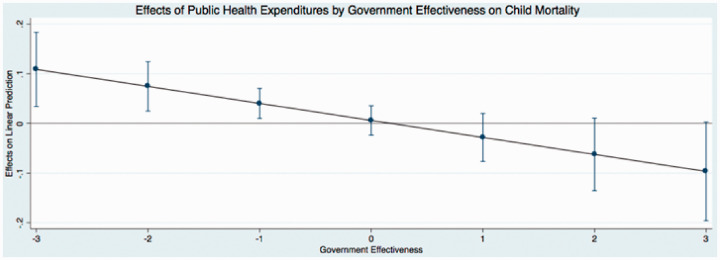
Predicted effects: effects of public health expenditures by government effectiveness on child mortality.

**Figure 5.**
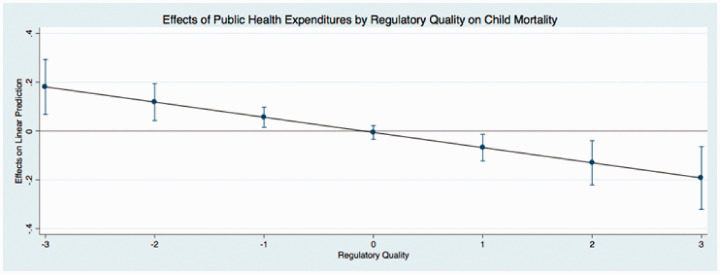
Predicted effects: effects of public health expenditures by regulatory quality on child mortality.

## Discussion and Conclusion

Using newly available, multidimensional governance data, this study demonstrates that increases in public spending on health may not lead to less child mortality if countries have low levels of governance.^10,18^ It is not entirely surprising that public medical care expenditures did not significantly predict child mortality, given the state of the current debate, although this finding diverges from previous studies.^18^ Also deviating from previous studies, governance alone has no impact on child health.^18^ Instead, the findings generally indicate that both public spending and governance together are essential to reduce child mortality cross-nationally.

Therefore, this study adds to the debate on the effectiveness of public medical care expenditures. The findings show that researchers who argue that government health spending is ineffective *and* those who claim that public medical care expenditures can continue to reduce child mortality are not entirely inaccurate. On the contrary, both sides of the debate were missing the ways in which governance, combined with health spending, can reduce child mortality. This study helps bring forward governance as a previously less studied piece of the puzzle concerning how nations may improve their health spending. It is evident that the main theoretical and practical contributions of this study reside with the need to look at both state structures of governance and health spending together, rather than as unconnected or isolated factors contributing to child health. In result, such insights are critical for debates on the effectiveness of government spending, but also for the continuing reduction of child mortality among developing nations.

Given that public medical care expenditures decrease child mortality more at higher levels than at lower levels of state governance, research should consider different types of state structures, economic and institutional, to understand the ability of a nation to increase child health and, most importantly, how economic and governance aspects of the state relate to one another.^19,26,29^ Moreover, social scientists should move beyond considering only political-economy theories, which tend to ignore how intranational processes influence health and well-being.^64^ Considering a state’s capacity for well-being will help us arrive at a more comprehensive understanding of what nations can do to mitigate their child mortality.^10^

At the minimum, increased attention must focus on designing and testing governance interventions in the health sector, such as centralization and tracking funding (which introduces extra checks on funds, where they go, and what they are used for).^65,66^ For example, the Ugandan government recently centralized health funding within its Ministry of Finance to National Medical Stores instead of sending health funds directly to health facilities.^66^ At the most extreme, nations can aim to uproot poor governance in its operations and can start by following through with anti-corruption policies and auditing systems.

Despite the importance of the main findings, we cannot generalize beyond the sample and time period used in this analysis (i.e., 1996–2012). Future researchers may aim to expand upon or duplicate this study when more data become available. It is important to note that the measurement of the governance indicators is inherently biased as a result of who collects it and how it is defined. Future operationalizations of governance should strive to employ evaluations that assess the functioning of a government from a less ethnocentric perspective. Researchers may also aim to understand how governance interacts with other development variables of interest, such as democracy, economic growth, or education rates.^19,29^

Still, to my knowledge, this study is to date the most comprehensive analysis of the relationships between governance and child mortality. Again, the findings of this study indicate that it is imperative for researchers to evaluate how different aspects of the state together impact human development in order to arrive at the most comprehensive understanding of well-being. The dynamics of governance, national health spending, and health outcomes are complexly interwoven and require theoretical and empirical integration rather than being considered isolated factors.

## Supplemental Material

sj-pdf-1-joh-10.1177_0020731420960972 - Supplemental material for Accountable Government Spending: A Cross-National Analysis of Child Mortality in Developing Nations Click here for additional data file. Supplemental material, sj-pdf-1-joh-10.1177_0020731420960972 for Accountable Government Spending: A Cross-National Analysis of Child Mortality in Developing Nations by Jamie M. Sommer in International Journal of Health Services

## References

[1] World Bank. *World Bank Indicators, Data*. Washington, DC: World Bank Group; 2016.

[2] Grekou C Perez R. Child mortality in sub-Saharan Africa: why public health spending matters. EconomiX (28) Working Papers from University of Paris West-Nanterre la Défense, EconomiX; 2014.

[3] Novignon J Olakojo SA Nonvignon J. The effects of public and private health care expenditure on health status in sub-Saharan Africa: new evidence from panel data analysis. Health Econ Rev. 2012; 2(1):22. 23232089 10.1186/2191-1991-2-22 PMC3533939

[4] Hanmer L Lensink R White H. Infant and child mortality in developing countries: analyzing the data for robust determinants. J Dev Stud. 2003; 40(1):101–118.

[5] Khan M. *Governance and Development: The Perspective of Growth-Enhancing Governan*ce. Tokyo, Japan: GRIPS Development Forum/National Graduate Institute for Policy Studies.

[6] Çevik S Taşar MO. Public spending on health care and health outcomes: cross-country comparison. J Bus Econ Finance. 2013; 2(4):82–100.

[7] Pritchett L. *Mind Your P’s and Q’s: The Cost of Public Investment Is Not the Value of Public Capital*. Washington, DC: World Bank; 1996.

[8] Anyanwu J Erhijakpor AE. Working Paper 92-Education Expenditures and School Enrolment in Africa: Illustrations from Nigeria and Other SANE Countries; 2007.

[9] Filmer D Pritchett L. The impact of public spending on health: does money matter? Soc Sci Med. 1999; 49(10):1309–1323. 10509822 10.1016/s0277-9536(99)00150-1

[10] Rajkumar AS Swaroop V. Public spending and outcomes: does governance matter? J Dev Econ. 2008; 86(1):96–111.

[11] Hitiris T Posnett J. The determinants and effects of health expenditure in developed countries. J Health Econ. 1992; 11(2):173–181. 10122977 10.1016/0167-6296(92)90033-w

[12] Makinen M Waters H Rauch M , et al. Inequalities in health care use and expenditures: empirical data from eight developing countries and countries in transition. Bull World Health Org. 2000; 78(1):55–65. 10686733 PMC2560608

[13] Gupta S Davoodi HR Tiongson E. Corruption and the Provision of Health Care and Education Services. Washington, DC: International Monetary Fund; 2000.

[14] Parsitau DS. Keep holy distance and abstain till he comes: interrogating a Pentecostal Church’s engagements with HIV/AIDS and the youth in Kenya. Africa Today. 2009; 56(1):45–64.

[15] Shandra JM Nobles JE London B Williamson JB. Multinational corporations, democracy and child mortality: a quantitative, cross-national analysis of developing countries. Soc Indic Res. 2005; 73(2):267–293.

[16] Pandolfelli LE Shandra JM. The African development bank, structural adjustment, and child mortality: a cross-national analysis of Sub-Saharan Africa. Int J Health Serv. 2013; 43(2):337–361. 23821909 10.2190/HS.43.2.i

[17] Factor R Kang M. Corruption and population health outcomes: an analysis of data from 133 countries using structural equation modeling. Int J Public Health. 2015; 60(6):633–641. 25994589 10.1007/s00038-015-0687-6

[18] Hu B Mendoza RU. Public health spending, governance and child health outcomes: revisiting the links. J Hum Dev Capab. 2013; 14(2):285–311.

[19] Lee CS. Income inequality, democracy, and public sector size. Am Sociol Rev. 2005; 70(1):158–181.

[20] Holmberg S Rothstein B Nasiritousi N. Quality of government: what you get. Ann Rev Polit Sci. 2009; 12:135–161.

[21] Lazarova EA. Governance in relation to infant mortality rate: evidence from around the world. Ann Public Cooperat Econ. 2006; 77(3):385–394.

[22] Campos NF Nugent JB. Development performance and the institutions of governance: evidence from East Asia and Latin America. World Dev. 1999; 27(3):439–452.

[23] Evans P Rauch JE. Bureaucracy and growth: a cross-national analysis of the effects of “Weberian” state structures on economic growth. Am Sociol Rev. 1999;64(5):748–765.

[24] Dawson A. State capacity and the political economy of child mortality in developing countries revisited: from fiscal sociology towards the rule of law. Int J Comp Sociol. 2010; 51(6):403–422.

[25] Kaufmann D Kraay A. The Worldwide Governance Indicators Project. Washington, DC: The World Bank Group; 2015.

[26] Evans PB. Predatory, developmental, and other apparatuses: a comparative political economy perspective on the third world state. Sociol Forum. 1989; 4(4):561–587.

[27] Shen C Williamson JB. Child mortality, women’s status, economic dependency, and state strength: a cross-national study of less developed countries. Soc Forces. 1997; 76(2):667–700.

[28] Shen C Williamson JB. Accounting for cross-national differences in infant mortality decline (1965–1991) among less developed countries: effects of women’s status, economic dependency, and state strength. Soc Indic Res. 2001; 53(3):257–288.

[29] Brady D Bostic A. Paradoxes of social policy: welfare transfers, relative poverty, and redistribution preferences. Am Sociol Rev. 2015; 80(2):268–298.

[30] Korpi W Palme J. The paradox of redistribution and strategies of equality: welfare state institutions, inequality, and poverty in the Western countries. Am Sociol Rev. 1998;63(5):661–687.

[31] Keep M. *Health Expenditure: International Comparisons*. London, England: House of Commons Library, Standard Notes SN/SG/2584; 2011.

[32] Pandolfelli LE Shandra J Tyagi J. The International Monetary Fund, structural adjustment, and women’s health: a cross-national analysis of maternal mortality in Sub-Saharan Africa. Sociol Q. 2014; 55(1):119–142.

[33] Gupta S Verhoeven M Tiongson ER. The effectiveness of government spending on education and health care in developing and transition economies. Eur J Polit Econ. 2002; 18(4):717–737.

[34] Devarajan S Swaroop V Zou HF. The composition of public expenditure and economic growth. J Monet Econ. 1996; 37(2):313–344.

[35] Kaufmann D Kraay A Mastruzzi M. The Worldwide Governance Indicators: A Summary of Methodology, Data and Analytical Issues. World Bank Policy Research Working Paper No. 5430; 2010.

[36] Ghobarah HA Huth P Russett B. The post-war public health effects of civil conflict. Soc Sci Med. 2004; 59(4):869–884. 15177842 10.1016/j.socscimed.2003.11.043

[37] Spiegel PB Checchi F Colombo S Paik E. Health-care needs of people affected by conflict: future trends and changing frameworks. Lancet. 2010; 375(9711):341–345. 20109961 10.1016/S0140-6736(09)61873-0

[38] Urdal H Che CP. War and gender inequalities in health: the impact of armed conflict on fertility and maternal mortality. Int Interact. 2013; 39(4):489–510.

[39] Kar D LeBlanc B. *Illicit Financial Flows From Developing Countries: 2002–2011*. Washington, DC: Global Financial Integrity; 2013.

[40] Villegas A Morales A Andersson N. Popular Perceptions of Corruption in Public Services: Key Findings of the First National Integrity Survey in Bolivia *[Unpublished manuscript]*. New York, NY: CIET International; 1998.

[41] Rothstein B Stolle D. The state and social capital: an institutional theory of generalized trust. Comparat Polit. 2008; 40(4):441–459.

[42] Akçay S. Corruption and human development. Cato J. 2006; 26:29.

[43] Gupta S Davoodi H Alonso-Terme R. Does corruption affect income inequality and poverty? Econ Govern. 2002; 25(3):23–45.

[44] World Health Organization. *Global Health Observatory (GHO) Data, Maternal Mortality*. Geneva, Switzerland: World Health Organization; 2015.

[bibr45-0020731420960972] 45. UNICEF. Health: The Big Picture. http://www.unicef.org/health/index_bigpicture.html. Published 2008. Accessed September 14, 2020.

[46] Bates RH. Prosperity and Violence: The Political Economy of Development. New York, NY: W.W. Norton & Co; 2010.

[47] Esman MJ Uphoff NT. Local Organizations: Intermediaries in Rural Development. Ithaca, NY: Cornell University Press; 1984.

[48] Saksena P Xu K Elovainio R Perrot J. Health services utilization and out-of-pocket expenditure at public and private facilities in low-income countries. World Health Rep. 2010;20: 20. 10.1111/j.1365-3156.2011.02894.x 22008480

[49] Transparency International UK. *Corruption: Cost for Developing Countries*. London, England: Transparency International: The Global Coalition Against Corruption; 2015.

[50] Halaby CN. Panel models in sociological research: theory into practice. Annu Rev Sociol. 2004; 30:507–544.

[51] Hsiao C. Analysis of Panel Data. Cambridge, England: Cambridge University Press; 2014.

[52] York R Rosa EA Dietz T. STIRPAT, IPAT and ImPACT: analytic tools for unpacking the driving forces of environmental impacts. Ecol Econ. 2003; 46(3):351–365.

[53] Shandra JM Nobles J London B Williamson JB. Dependency, democracy, and infant mortality: a quantitative, cross-national analysis of less developed countries. Soc Sci Med. 2004; 59(2):321–333. 15110423 10.1016/j.socscimed.2003.10.022

[54] Jorgenson AK Clark B. The economy, military, and ecologically unequal exchange relationships in comparative perspective: a panel study of the ecological footprints of nations, 1975–2000. Soc Probl. 2009; 56(4):621–646.

[55] Jorgenson AK Dick C Mahutga MC. Foreign investment dependence and the environment: an ecostructural approach. Soc Probl. 2007; 54(3):371–394.

[56] Rice J. Material consumption and social well-being within the periphery of the world economy: an ecological analysis of maternal mortality. Soc Sci Res. 2008; 37(4):1292–1309. 19227704 10.1016/j.ssresearch.2008.05.006

[57] Vanhanen T. Global Inequality: As a Consequence of Human Diversity: A New Theory Tested by Empirical Evidence. London, England: Ulster Institute for Social Research; 2014.

[58] Lake DA Baum MA. The invisible hand of democracy: political control and the provision of public services. Comp Polit Stud. 2001; 34(6):587–621.

[59] Zweifel TD Navia P. Democracy, dictatorship, and infant mortality. J Democr. 2000; 11(2):99–114.

[60] Foster G Williamson J. A review of current literature on the impact of HIV/AIDS on children in Sub-Saharan Africa. AIDS. 2000; 14(3):275–284. 11086871

[61] Scanlan SJ. Gender, development, and HIV/AIDS: implications for child mortality in less industrialized countries. Int J Comp Sociol. 2010; 51(3):211–232.

[62] Schultz TP. Human capital, family planning, and their effects on population growth. Am Econ Rev. 1994; 84(2):255–260.

[63] London B Ross RJ. The political sociology of foreign direct investment. Int J Comp Sociol. 1995; 36(3):198–218.

[64] London B Williams BA. Multinational corporate penetration, protest, and basic needs provision in non-core nations: a cross-national analysis. Soc Forces. 1988; 66(3):747–773.

[65] Vian T. Review of corruption in the health sector: theory, methods and interventions. Health Policy Plann. 2008; 23(2):83–94. 10.1093/heapol/czm048 18281310

[66] Kohler JC. Fighting Corruption in the Health Sector: Methods, Tools and Good Practices. New York, NY: United Nations Development Programme; 2011.

