# Supplementary material for: Accountable Government Spending: A Cross-National Analysis of Child Mortality in Developing Nations
Source: Int J Health Serv. 2020 Oct 5;52(1):73–88. doi: 10.1177/0020731420960972 (PMC8592112; doi:10.1177/0020731420960972)
Supplement: sj-pdf-1-joh-10.1177_0020731420960972 - Supplemental material for Accountable Government Spending: A Cross-National Analysis of Child Mortality in Developing Nations [file sj-pdf-1-joh-10.1177_0020731420960972.pdf]

Supplementary Files

1). Afghanistan (2005, 2007–2011); Angola (1998–2003, 2006–2011); Armenia (2002–2011); Azerbaijan (1996–1998, 2007–2011); Bangladesh (2000–2010); Belarus (1996–2007, 2010, 2011); Belize (1996, 1997, 1999–2011); Benin (2000–2006, 2011), Bhutan (1997–2008); Bolivia (2001–2004, 2006); Botswana (2005–2009); Burkina Faso (2001, 2003–2011); Cabo Verde (2008–2011); Cambodia (2001–2011); Cameroon (1997, 1998); Colombia (2002, 2007–2011); Congo, Dem. Rep. (2002, 2008–2010); Congo, Rep. (2000–2007); Costa Rica (2007–2011); Dominican Republic (2004, 2005 and 2007–2010); Egypt, Arab Rep. (1996, 2001, 2003, 2004, 2009–2011); El Salvador (2001–2011); Ethiopia (1996, 1997, 2000–2010); Fiji (2003, 2004); The Gambia (2000–2008); Georgia (1996, 1997, 2000–2011); Ghana (2000–2009); Guatemala (1999–2002, 2004–2011); Guinea (1997, 1998); India (1996, 1997, 1999–2008); Indonesia (1996, 2001–2008); Iran (1999–2007); Jamaica (2009–2011); Kenya (1998–2009); Kyrgyz Republic (1996, 1999, 2000, 2005–2011); Lao PDR (2005–2011); Lesotho (1996–2001); Liberia (2006, 2009, 2011); Madagascar (1999–2009); Malaysia (1998, 1998–2011); Maldives (2002–2005); Mali (1999, 2000, 2003–2012); Mauritius (2008–2011); Mexico (1996–1999); Moldova (1996, 1997, 1999–2011); Mongolia (1996–2011); Morocco (2001–2011); Mozambique (2009–2011); Namibia (1996–2010); Nepal (1999–2012); Nicaragua (1996, 1997, 2000–2002, 2004–2008, 2010); Niger (2004–2006); Pakistan (1996, 2002, 2006–2012); Panama (1999, 2000); Paraguay (2004–2011); Peru (1998–2011); Philippines (1998–2009); Rwanda (2007–2009, 2011, 2012); Sao Tome and Principe (2003–2005, 2007–2011); Senegal (2008–2011); South Africa (1999–2009); Sri Lanka (2001–2006, 2008–2011); Suriname (2001, 2002, 2005, 2007); Tajikistan (1997, 1999–2003); Tanzania (2008–2010); Thailand (2002, 2004–2011); Togo (2003–2011); Tunisia (1996–2011); Uganda (1997, 2000–2011); Ukraine (1998–2011); and Venezuela, RB (1996–2004); Zambia (1998, 2000–2002, 2004, 2005, 2007–2010).

2). Three variables (water/sanitation, GDP, and measles immunizations) exceeded a VIF value of 2.5, which indicates potential problems with multicollinearity. To address these high correlations, I ran a series of models with only one of the highly correlated predictors. The results are similar to the findings presented, indicating no potential problems with multicollinearity.

3). Public medical care expenditures as a percentage of GDP is included in the analysis rather than total health expenditures or private health expenditures because it measures only health funds allocated by the central government for public use. Lumping together both private and public is not of theoretical importance in this specific study because the main interest concerns the states' prioritization of health funds, rather than its influence or coordination with private funders and companies. Moreover, this study builds upon previous research by Dawson (2010) and Rajkumar and Swaroop (2008), which holds that this particular measure is of direct importance to child mortality compared to per capita or total expenditure operationalization due to the central government's economic strength (10, 24).

4). Following previous studies (18), it is important to check for issues with endogeneity when assessing the relationship among public health spending, governance, and child mortality (18). Endogeneity can be problematic when one of the independent variables is jointly determined with the dependent variable being explained (67, 68). In this case, endogeneity may be biasing the estimates because governance and health expenditures are not randomly assigned and may be linked to other factors external or internal to a

nation. For instance, a nation might increase its health expenditures because it has very high child mortality levels. If this is the case, then the regression equation would be capturing the selection of levels health spending as well as the effects of prioritizing health spending in one parameter (68). To test for this, researchers usually find a variable to be an instrument that is correlated with the independent variable under question but not the dependent variable. After selecting an instrument, I ran the Davidson–MacKinnon test of exogeneity. The results are inconclusive, though the main findings remain consistent to those reported, suggesting that endogeneity is not biasing the coefficients.
